# A Kir6.2 Pore Mutation Causes Inactivation of ATP-Sensitive Potassium Channels by Disrupting PIP_2_-Dependent Gating

**DOI:** 10.1371/journal.pone.0063733

**Published:** 2013-05-20

**Authors:** Jeremy D. Bushman, Qing Zhou, Show-Ling Shyng

**Affiliations:** Department of Biochemistry and Molecular Biology, Oregon Health and Science University, Portland, Oregon, United States of America; Universidad Autonoma de San Luis Potosi, Mexico

## Abstract

In the absence of intracellular nucleotides, ATP-sensitive potassium (K_ATP_) channels exhibit spontaneous activity via a phosphatidylinositol-4,5-bisphosphate (PIP_2_)-dependent gating process. Previous studies show that stability of this activity requires subunit-subunit interactions in the cytoplasmic domain of Kir6.2; selective mutagenesis and disease mutations at the subunit interface result in time-dependent channel inactivation. Here, we report that mutation of the central glycine in the pore-lining second transmembrane segment (TM2) to proline in Kir6.2 causes K_ATP_ channel inactivation. Unlike C-type inactivation, a consequence of selectivity filter closure, in many K^+^ channels, the rate of inactivation in G156P channels was insensitive to changes in extracellular ion concentrations or ion species fluxing through the pore. Instead, the rate of G156P inactivation decreased with exogenous application of PIP_2_ and increased when PIP_2_-channel interaction was inhibited with neomycin or poly-L-lysine. These findings indicate the G156P mutation reduces the ability of PIP_2_ to stabilize the open state of K_ATP_ channels, similar to mutations in the cytoplasmic domain that produce inactivation. Consistent with this notion, when PIP_2_-dependent open state stability was substantially increased by addition of a second gain-of-function mutation, G156P inactivation was abolished. Importantly, bath application and removal of Mg^2+^-free ATP or a nonhydrolyzable analog of ATP, which binds to the cytoplasmic domain of Kir6.2 and causes channel closure, recover G156P channel from inactivation, indicating crosstalk between cytoplasmic and transmembrane domains. The G156P mutation provides mechanistic insight into the structural and functional interactions between the pore and cytoplasmic domains of Kir6.2 during gating.

## Introduction

Inwardly rectifying potassium (Kir) channels are expressed in a wide variety of cell types where they regulate membrane excitability in response to diverse signals [1]. Among them, ATP-sensitive potassium (K_ATP_) channels composed of Kir6.2 and sulfonylurea receptor 1 (SUR1) play a critical role in controlling insulin secretion and neuronal excitability [2]–[4]. Like all Kir channels, K_ATP_ channels are activated by membrane phosphoinositides, especially phosphatidyl-inositol-4,5-bisphosphates (PIP_2_) [5]–[7]. PIP_2_ binds to the cytoplasmic domain of Kir6.2 and opens the channel; this gating process underlies the channel’s intrinsic open probability. Intracellular ATP, which binds overlapping but non-identical site as PIP_2_, competes with PIP_2_ functionally and closes the channel (reviewed in [8]). The majority of evidence to date suggests a model in which a gate located near the helix bundle crossing where the four inner helices converge, commonly referred to as the “lower” gate, is sensitive to PIP_2_ and ATP regulation [9]–[11]. In addition, a gate located near the selectivity filter, referred to as the “upper” gate, controls the ligand-independent fast gating observed in single channel kinetics [12].

A central question in Kir channel gating is how ligand interaction with the cytoplasmic domain of the channel leads to opening or closing of the channel. There is considerable evidence that opening of the channel by activating ligands is associated with rotation and bending of the inner helix (TM2) and widening of a lower gate [13]–[17] (also see review [18]). Bending of TM2 requires structural flexibility of the alpha helix. Early studies of K^+^ channels such as MthK and Kv channels have led to a glycine hinge hypothesis whereby a highly conserved glycine in the middle of TM2 is thought to provide the flexibility that allows the helix to bend during gating [19]. Interestingly, studies of Kir3.4 channels, which are activated by Gβγ, have shown that substitution of the central glycine with proline (G175P) nearly eliminated basal channel activity and this effect was thought to support the hinge hypothesis. However, later studies found that substituting the glycine with other amino acids in Kir3 did not eliminate channel activity, although it did impact single channel gating kinetics that was explained by interactions of substituting amino acids with residues in the selectivity filter and the pore helix [20], [21]. These results argue that the central glycine, rather than serving as a hinge, is necessary to prevent constraining interactions with critical residues in its vicinity [20]. In Kir6.2, mutation of the equivalent central glycine residue to an arginine (G156R) has been identified in patients with congenital hyperinsulinism. Our previous study showed that the G156R mutation abolishes channel activity and this gating defect is overcome by a second-site mutation N160D approximately one helical turn down TM2 [22]. In the G156R/N160D double mutant the two mutant residues interact electrostatically to recover ion conduction and channel gating by intracellular ligands. The study led us to conclude that as in GIRK channels, the glycine corresponding to the hinge in MthK is not essential for gating in K_ATP_ channels.

In this work, we report the discovery that mutation of the central glycine in Kir6.2 to proline, G156P, causes an unexpected rapid inactivation phenotype. We present evidence that this pore mutation decreases channel open state stability by disrupting a gate controlled by PIP_2_ which binds the cytoplasmic domains of the channel. Furthermore, we show that inactivation caused by the G156P mutation can be recovered by exposure to the inhibitory ligand ATP followed by washout of ATP, implicating an ATP-induced conformational change that resets the inactivated TM2 to allow it to couple to the PIP_2_-controlled gate. These findings provide insight to the structural mechanisms that govern K_ATP_ channel activity.

## Materials and Methods

### Molecular Biology

Rat Kir6.2 and hamster SUR1 cDNAs were in pUNIV vector to enhance surface expression [23]. Mutagenesis was performed using the QuikChange site-directed mutagenesis kit (Stratagene), and mutations were confirmed by sequencing. Mutant clones from two or more independent PCR reactions were analyzed to avoid false results caused by undesired mutations from PCR.

### Chemiluminescence Assay

COSm6 cells were maintained in DMEM with 10% fetal bovine serum and 1% Penicillin/Streptomycin. Cells ∼70% confluent on 35 mm dishes were transfected with cDNA of SUR1 (0.6 µg) and Kir6.2 (0.4 µg) using FuGene®6 (Roche). At 48–72 hr after transfection, cell surface expression levels of WT and mutant channels were assessed by a quantitative chemiluminescence assay using a SUR1 that was tagged with a FLAG epitope (DYKDDDDK) at the N-terminus (f-SUR1), as described previously [24]. Cells were fixed with 2% paraformaldehyde for 20 min at 4°C. Fixed cells were pre-blocked in phosphate-buffered saline (PBS) plus 0.1% bovine serum albumin (BSA) for 30 min, incubated with the M2 mouse monoclonal anti-FLAG antibody (10 µg/ml, Sigma) for 1 h to label f-SUR1, washed 3×20 min in PBS plus 0.1% BSA, incubated with a horseradish peroxidase-conjugated anti-mouse secondary antibody (Jackson ImmunoResearch, Inc., 1∶1000 dilution) for 30 min, and washed again 4×30 min in PBS plus 0.1% BSA. Chemiluminescence of each dish was quantified in a TD-20/20 luminometer (Turner Designs) after 10 sec of incubation in Power Signal enzyme-linked immunosorbent assay luminol solution (Pierce). All steps after fixation were carried out at room temperature. The signal observed in untransfected control cells was ∼11% of that observed in cells transfected with WT channels and was subtracted as background for data analysis.

### Electrophysiology

Patch-clamp recordings were performed in the inside-out configuration. COSm6 cells were transfected with cDNA encoding WT or mutant channel proteins as well as cDNA for the green fluorescent protein to help identify transfected cells. Patch-clamp recordings were made 36–72 h post-transfection. Micropipettes were pulled from non-heparinized Kimble glass (Fisher) with resistance of 0.5–2 megaohms. The bath (intracellular) and pipette (extracellular) solution Kint had the following composition: 140 mM KCl, 10 mM K-HEPES, 1 mM K-EGTA, 1 mM K-EDTA, pH 7.3. EDTA was included in all solutions to prevent channel rundown. ATP and AMP-PNP were added as the potassium salt (Sigma-Aldrich). PIP_2_ (Sigma) was reconstituted in Kint at 5 mM; aliquots were stored at −20°C until use and then diluted in Kint/EDTA and sonicated for 15 minutes in ice water prior to use. Oleoyl-CoA was dissolved in Kint at 5 µM final concentration. For ion substitution experiments, KCl was replaced with either 5 mM KCl plus 135 mM N-Methyl-D-Glucamine (NMDG), 300 mM KCl, or 150 mM RbCl. Command potentials varied and are stated in the figure legends as membrane potential (negative of command potential in inside-out patch). When ATP sensitivity was reduced, base-line currents were obtained using 10 mM BaCl_2_ block at +50 mV membrane potential.

To achieve rapid perfusion, a home-made piezo-driven device was used to move a theta pipette flowing Kint and Kint+ATP solutions on an excised patch. Open-tip current response 10–90% rise times to high versus low K^+^ solutions was less than 5 ms, whereas WT and mutant K_ATP_ channel patch current rise times were typically 50–100 ms (data not shown), consistent with previous rapid perfusion measurements [5]. Slow-to-form membrane patch seals with >200 ms rise times coincided with observing highly variable inactivation rates within the same patch, and these data were discarded. To measure single channel conductance, currents were recorded using borosilicate glass (Sutter Instruments) electrodes coated with Sylgard and polished with a microforge (Narishige) to produce bath resistances of 8.0–12.0 megaohms. Currents were acquired at 50 kHz with an applied analog 4-pole low-pass Bessel filter with a cutoff frequency of 5–10 kHz. Recordings were filtered again offline using an 8-pole Bessel filter with a cutoff of 2 kHz prior to analysis and presentation.

### Data Analysis

Data were analyzed and displayed using pCLAMP software (Axon Instruments), Microsoft Excel and Origin programs. Statistical analysis was performed using the following: for current amplitudes, we used independent two populations, two-tailed Student’s *t*-test; for voltage-dependence, ion substitution and neomycin or poly-lysine sensitivity comparisons, we used one-way analysis of variance (ANOVA) with Tukey *post-hoc* test.

## Results

### Central Glycine Mutation in Kir6.2 Inactivates K_ATP_ Channels

**Figure 1:**
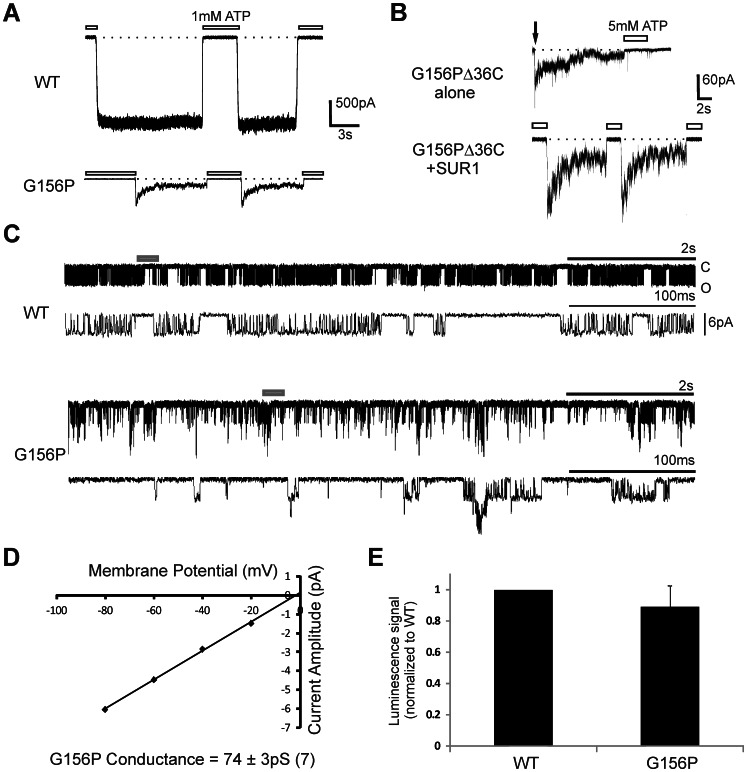
Inactivation Phenotype of G156P Channels. (A) Sample traces of voltage clamp inside-out patch recordings containing WT or G156P mutant channels. Dashed lines show current baseline. (B) Sample traces of channels formed by Kir6.2 G156PΔ36C alone or co-assembled with SUR1 (n = 4 and 5 patches, respectively). In A and B, membrane potential was −50 mV. Downward arrow indicates time of patch excision. (C) Sample traces of inside-out recordings at −80 mV from one (WT) or several (G156P) channels. Dark gray bar denotes region displayed with increased time resolution below. (D) A representative current-voltage relation for G156P single channels. Values are the mean of Gaussian fits to identifiable peaks in an amplitude histogram for a given potential (n = 7 patches). Conductance is measured from the slope of the line. (E) Surface expression measured from quantitative chemiluminescence assay; see methods for details.

In an effort to further understand the structural and functional role of the central glycine in Kir6.2 following our initial study of the G156R disease mutation [22], we substituted G156 with other amino acids and examined the resulting channels by inside-out patch-clamp recording. Many mutations resulted in non-functional channels (Table S1). Among the mutants that gave rise to detectable channel activity, the G156P stands out for a pronounced phenotype distinct from wild-type (WT) channels (Table S1). In K^+^ solution containing 1 mM EDTA that minimizes channel current rundown, WT K_ATP_ channels produced stable spontaneous current amplitudes reflecting an ensemble intrinsic channel *P_o_* of ∼0.5 in an excised inside-out patch in the absence of nucleotides (Fig. 1A). In contrast, the G156P mutant exhibited a rapid decay in channel activity under the same recording condition (Fig. 1A). The residual current was inhibited by exposure to ATP. Interestingly, however, upon subsequent removal of ATP, channel activity recovered and underwent another round of rapid decay (Fig. 1A; see below). The gating phenotype resembles ‘inactivation’ we and others previously reported in several mutant channels [25]–[28]. These include channels with Kir6.2 mutations in the cytoplasmic domain at the Kir6.2 subunit-subunit interface, as well as a channel that carries a SUR1 mutation, E128W, in the second cytoplasmic loop of the first transmembrane domain [29]. Note although G156C, G156N and G156T also showed some current decay, the decays occurred at a much slower time scale and did not always recover after ATP exposure and washout. We therefore focused our attention on the G156P mutation.

To investigate the mechanism by which G156P-Kir6.2 causes inactivation, we first tested the possibility that the mutation disrupts interactions with SUR1. SUR1 elevates the Kir6.2 *P_o_* from ∼0.1 to ∼0.5 by enhancing channel interaction with PIP_2_, and interfering with Kir6.2-SUR1 coupling could lead to inactivation, as observed in E128W-SUR1 mutant channels [29]. If G156P-induced inactivation is intrinsic to Kir6.2, inactivation should still be observed in channels formed by Kir6.2 alone. Full-length Kir6.2 cannot express at the cell surface without SUR1 shielding an ER retention signal [30]. We therefore examined a Kir6.2 variant in which the C-terminal 36 amino acids where the ER retention signal resides have been deleted, referred to as Kir6.2Δ36C [31]. In contrast to WT Kir6.2Δ36C channels which displayed stable currents as reported previously (not shown; [26]), the G156P-Kir6.2Δ36C channel expressed in the absence of SUR1 produced currents which inactivated rapidly similar to that seen with G156P-Kir6.2/SUR1 channels (Fig. 1B). This result provides evidence that the mutant Kir6.2 tetramer alone is sufficient to confer inactivation. However, these currents did not recover with the ATP exposure and washout protocol that effectively recovered inactivated G156P-Kir6.2/SUR1 channels. When G156P-Kir6.2Δ36C was co-expressed with SUR1, inactivated mutant channels were again re-activated by ATP. These results suggest SUR1 is involved in mediating the ATP reactivation effect, possibly by increasing the *P_o_* (i.e. PIP_2_ interaction) and/or ATP sensitivity of the channel pore [31] (see Discussion).

Compared to WT channels, G156P-Kir6.2/SUR1 channels show lower peak current amplitudes upon patch excision (Fig. 1A). When G156P channel inactivation has reached a steady state, single channel openings could often be resolved. These openings occurred in low frequency and were usually separated by long-lived periods of inactivity, although bursts of activity were occasionally observed (Fig. 1C). Single mutant channel conductance estimated from these recordings was ∼74 pS (Fig. 1D), a value similar to our previous report for WT channels using identical conditions [22], indicating the G156P mutation does not affect K^+^ flux through the pore. To determine if the reduced macroscopic peak current is due to reduced mutant channel surface expression, we quantified surface channel expression using a chemiluminescence assay. In this experiment, equal amounts of WT or G156P-Kir6.2 cDNA was co-transfected with cDNA encoding FLAG-epitope tagged SUR1 (f-SUR1). As surface expression of both Kir6.2 and f-SUR1 requires co-assembly of the two subunits into an octameric channel complex, the amount of surface f-SUR1 labeled by anti-FLAG antibody and detected by a chemiluminescence enzyme reaction reflects the abundance of surface channels [30]. Luminescence measured from immuno-labeled surface G156P channels was similar to WT (Fig. 1E). Thus, reduced ensemble current amplitudes were not due to changes in conductance or channel expression, and were likely a result of channel inactivation that has occurred at the time of patch excision.

### ATP Reactivates G156P Channels in a Hydrolysis-independent Manner

**Figure 2:**
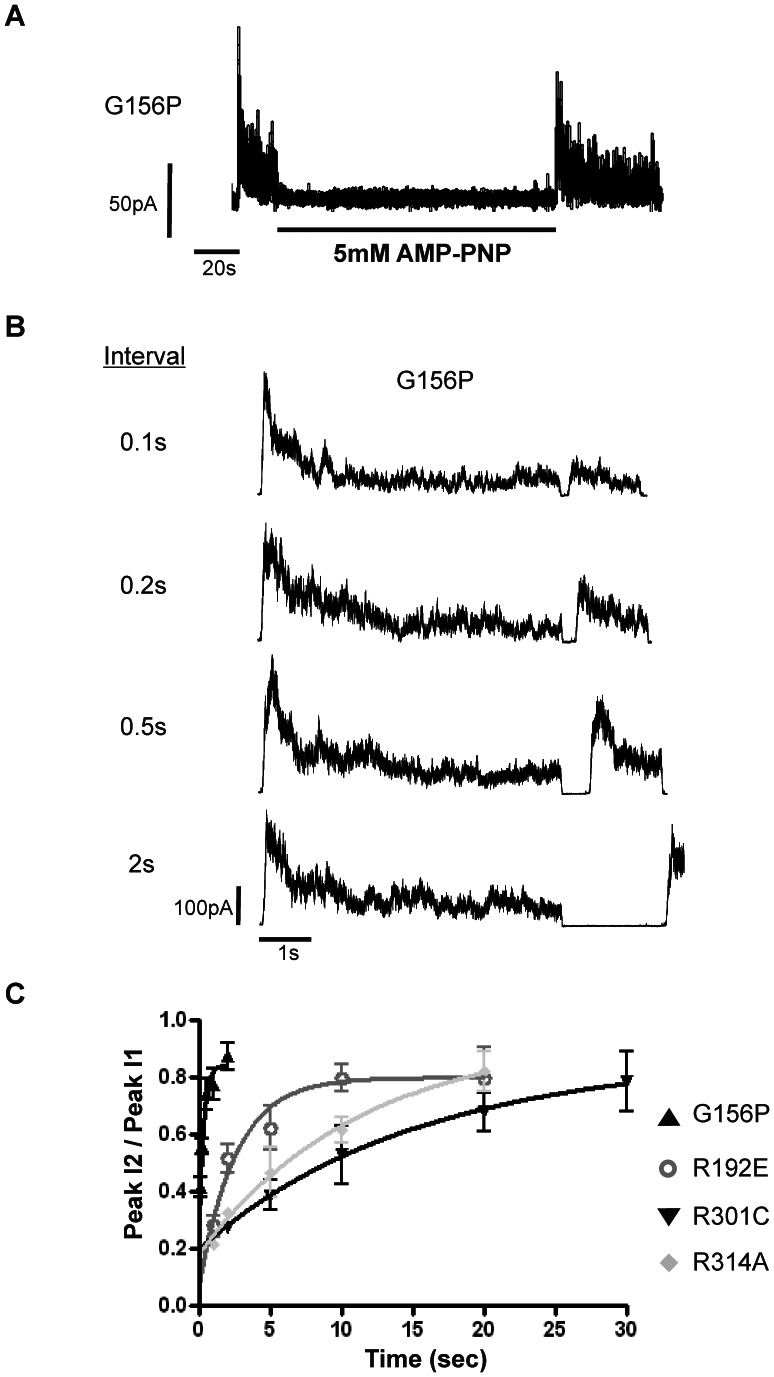
ATP recovers channels from inactivation. (A) A representative recording of G156P channels shows that exposure to 5 mM AMP-PNP followed by washout also recovered channels from inactivation. (B) Sample traces from the same patch subjected to the paired pulse protocol. Between each pair, G156P channels were exposed to 5 mM ATP for 30 seconds to induce maximal recovery. Then channels were activated to the first peak (Peak I_1_), and allowed to inactivate to reach a plateau, followed by a short interval of ATP exposure (5 mM) and removal to reach a second peak (Peak I_2_). Intervals were extended until the second peak reached near maximum. (C) Plot of reactivation (expressed as Peak I_2_/Peak I_1_) time course for G156P and other inactivation mutations. In a given patch, 2–3 trials for each interval shown in the plot were tested. Data for each mutation is from 3–5 patches. Note in some patches, mild rundown occurred after extended period of recording even in Kint/1 mM EDTA. This explains why the inactivation recovery did not reach 100% (i.e. Peak I_2_/PeakI_1_ = 1). We chose to not correct for rundown in the plot shown since the extent of rundown is similar for all mutations.

As shown in Fig. 1A, inactivated G156P channels are reactivated upon exposure and subsequent removal of ATP. ATP is known to have both inhibitory and stimulatory effects on K_ATP_ channels. Inhibition is mediated by interaction with Kir6.2 and does not require ATP hydrolysis [31]; whereas stimulation is mediated by interaction with SUR1 or replenishment of membrane PIP_2_, both of which require Mg^2+^ and ATP hydrolysis [6], [32], [33]. We tested whether reactivation of G156P channels by ATP requires nucleotide hydrolysis. Exposure of inactivated G156P channels to AMP-PNP, a non-hydrolyzable ATP analog, caused channel inhibition; upon subsequent removal of AMP-PNP, inactivated channels were reactivated (Fig. 2A). This result indicates that the ATP-induced reactivation of G156P is a consequence of ATP binding and does not require ATP hydrolysis, similar to that reported previously for the Kir6.2 cytoplasmic domain inactivation mutants [26].

To further characterize the reactivation effect of ATP, we compared the reactivation rate of G156P to that of previously identified Kir6.2 inactivation mutations using a paired pulse protocol (Fig. 2B). For these experiments, the patches were first subjected to ATP exposure (5 mM) and washout until peak currents reached a maximum to avoid complications arising from variable extent of inactivation at the time of excision. Solutions with or without ATP were then exchanged across the inside-out patch using rapid perfusion (see Methods) to expose channels to ATP with increasing durations. We observed greater recovery of channel activity upon subsequent ATP removal as the ATP exposure time increased (Fig. 2B). Interestingly, in comparison to previously reported Kir6.2 cytoplasmic domain inactivation mutants, R192E, R301C and R314A, the G156P mutant had a remarkably short reactivation time course requiring only a 2-second exposure to ATP to reach maximal reactivation (Fig. 2C; see Discussion).

### G156P Inactivation does not Exhibit Properties of C-type Inactivation

In GIRK channels, substitutions at the central glycine have been shown to interact with residues in the selectivity filter and the pore loop to alter gating kinetics [20], [21]. This prompted us to ask if the inactivation observed in the G156P-Kir6.2 mutant differs from that described previously for other Kir6.2 mutants involving a loss of integrity of the cytoplasmic domain structure [25], [26] and instead results from closure of the selectivity filter gate. This latter mechanism would be analogous to the C-type inactivation well studied in potassium channels such as Kv and KcsA. In C-type inactivation, opening of the lower gate near the bundle crossing triggers closure of the upper gate near the selectivity filter [34]. C-type inactivation has several distinctive features. The rate of C-type inactivation is dependent on factors that influence ionic occupancy of the pore, such as the conducting ion species and the external K^+^ concentration [35], [36]. Ions that bind more tightly to the selectivity filter or an increase in the external K^+^ concentration are thought to slow down selectivity filter closure following channel activation thus decreasing the inactivation rate. We tested these salient C-type inactivation features in the G156P-Kir6.2 mutant.

**Figure 3:**
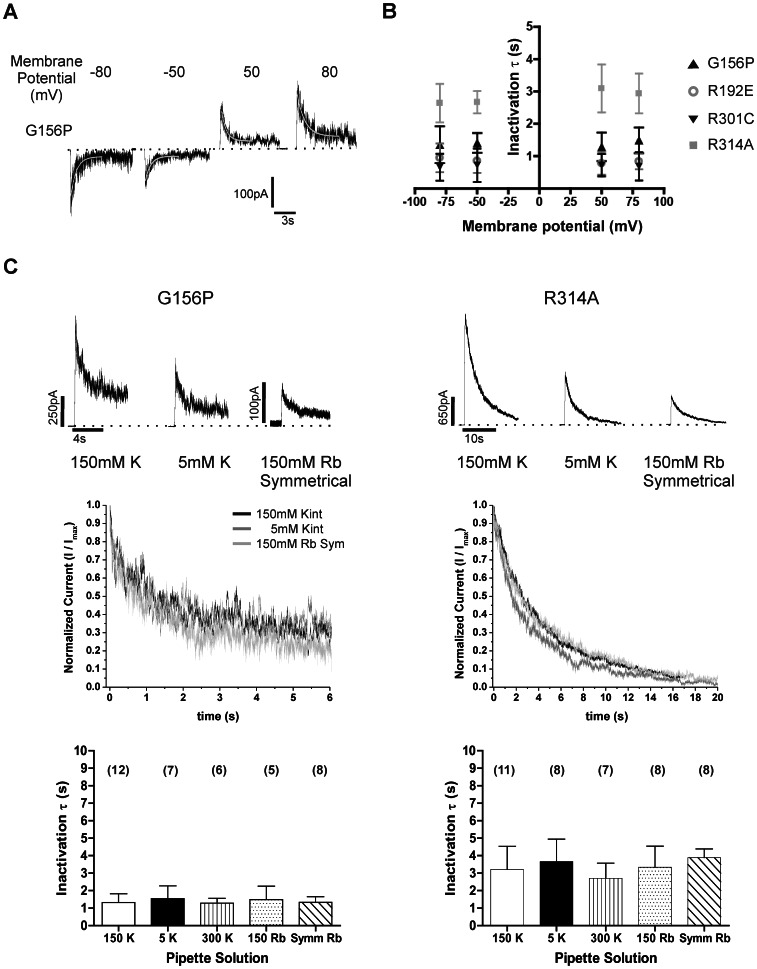
The inactivation rate of G156P channels is independent of membrane potential, ion concentrations or ion species. (A) G156P channels were re-activated in an inside-out patch by switching from Kint with 5 mM ATP to Kint alone at different membrane potentials. Current traces from the same patch were fit with a mono-exponential decay function (gray line). (B) Summary graph of inactivation time constants (τ) derived from quantitative decay fits for different inactivation mutations. Previously characterized mutations R192E [26], R301C [25] and R314A [26] are located in the cytoplasmic domain. Each data point represents mean ± S.E.M. of 5–10 patches. No significant difference was observed in the decay time constant between different membrane potentials for any of the inactivation mutations tested (one-way ANOVA). (C) Currents from G156P (*left column*) and R314A (*right column*) channels under different ion conditions were analyzed. With the exception of Symm Rb which has 150 mM Rb^+^ in both pipette (extracellular) and bath (intracellular) solutions, all others have 150 mM K^+^ in the bath solution. *Top*, sample traces from different G156P and R314A channel inside-out patches using symmetrical 150 mM K^+^, 5 mM extracellular/150 mM intracellular K^+^, or symmetrical 150 mM Rb^+^ ion conditions. *Middle*, plot of current decay time course under different conditions normalized to the maximal currents at time 0 (I_max_). *Bottom*, Summary bar plot of mean inactivation time constant τ ± S.E.M. for G156P and R314A under the different ion conditions tested. Currents were recorded at +80 mV membrane potential. Number of patches for each condition is shown above the bar in brackets. There is no statistically significant difference in inactivation rates measured under different conditions (one-way ANOVA).

To measure inactivation rate consistently within a given patch, G156P channel currents were allowed to reach peak amplitudes by repeated ATP exposure and washout after patch excision, and the patch was exposed to ATP for 5 seconds in between two measurements to ensure full reactivation (Fig. 2C). This protocol gave rise to reproducible peak currents and inactivation rate. Using this protocol, we first measured the inactivation rate of G156P in the same patch under symmetrical Kint with different command potentials ranging from −80 mV to +80 mV. Similar measurements were made in three other inactivation mutants, R192E, R301C and R314A (with the ATP exposure time in between two measurements adjusted accordingly based on results shown in Fig. 2C). As shown in Fig. 3A, the G156P current decayed to an initial plateau along a single exponential time course. We note that in many cases the plateau steady-state currents continued to decrease but rarely reached baseline, and optimal curve fits were obtained when the current initially reached a plateau. By this fitting rationale, no significant difference was observed in the decay time constant between different membrane potentials for any of the inactivation mutations tested (Fig. 3B). Control experiments showed that WT channel activity is stable in the same range of command potentials (see Fig. 1A).

We then measured the inactivation rate of G156P channels under different ionic conditions. Outward (+80 mV) current decay for G156P did not vary under low or high K^+^ conditions, or when K^+^ was replaced with Rb^+^ (Fig. 3C, *left*). Rb^+^ decreases single channel conductance in WT channels, probably from binding more tightly than K^+^ to the filter [37], [38] and has been shown to slow the C-type inactivation rate in other potassium channels [39]. Similarly, inactivation rate in the R314A mutant did not change significantly when the K^+^ concentration was altered or when K^+^ was replaced with Rb^+^ (Fig. 3C, *right*). As controls, WT channels were recorded under the same conditions and found to have stable currents within the time course examined (not shown). These results indicate the inactivation mechanism of G156P channels differs from C-type inactivation seen in *Shaker* K^+^ or KcsA channels, and more likely resembles the mechanism of other Kir6.2 cytoplasmic domain mutations.

### G156P Inactivation Rate depends on PIP_2_-channel Interaction

**Figure 4:**
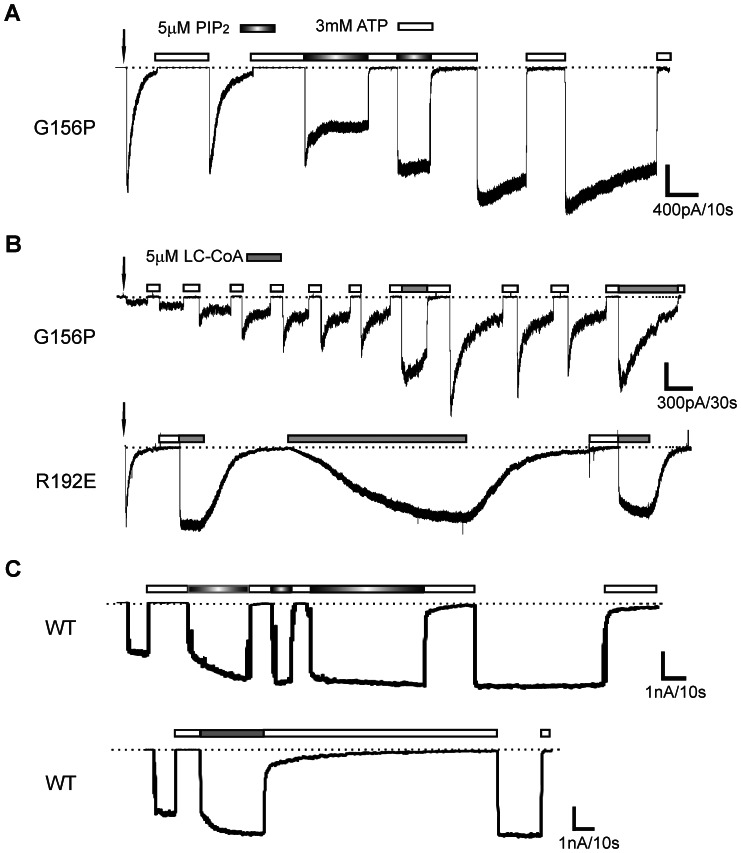
G156P inactivation is antagonized by PIP_2_ or LC-CoA. (A) Sample trace of G156P channels reactivated by 3 mM ATP into Kint or Kint with 5 µM PIP_2_. Downward arrow indicates time of patch excision. (B) Sample traces of G156P or R192E channels exposed to ATP to recover activity followed by either Kint or Kint with 5 µM oleoyl co-enzyme A (LC-CoA). G156P channel current decayed in the presence of LC-CoA; R192E activity was recovered by LC-CoA without ATP exposure. Note effect of LC-CoA was reversible. (C) Sample traces of WT channels exposed to PIP_2_ or LC-CoA. As reported previously [7], [43], exposure to PIP_2_ or LC-CoA increased channel activity and rendered channels less sensitive to ATP inhibition as evident from the slower response time to 3 mM ATP (irreversible in the case of PIP_2_ and reversible in the case of LC-CoA).

Phosphoinositides, in particular PIP_2_, are necessary for the intrinsic activity of Kir channels by controlling a gate or gates near the cytoplasmic end of the pore [13]–[17]. Our previous studies have shown that inactivation caused by mutations in the cytoplasmic domain of Kir6.2 thought to disrupt Kir6.2-Kir6.2 interactions could be reversed or recovered by exogenous PIP_2_ applied to the cytoplasmic face of the channel [25], [26]. If G156P mutation disrupts PIP_2_-dependent open state stability, then exogenous PIP_2_ should enhance intrinsic activity and slow down or prevent inactivation. We exposed excised patches containing G156P channels to PIP_2_ and found that PIP_2_ indeed stabilized channel activity, i.e. reduced or eliminated current decay, and increased peak current amplitudes after exposure to ATP and subsequent washout of ATP (Fig. 4A). Long-chain CoA (LC-CoA; oleoyl CoA was used in this study) which also activates K_ATP_ via a mechanism similar to PIP_2_ [40], [41] likewise antagonized channel inactivation (Fig. 4B). WT channels were exposed to PIP_2_ and LC-CoA to serve as controls (Fig. 4C). As reported in many previous studies, both PIP_2_ and LC-CoA increased WT channel activity and rendered channels less sensitive to ATP inhibition [5], [7], [29], [40]–[43].

When membrane patches were excised, initial channel current often varied in amplitude and inactivation rate (compare G156P current traces in Fig. 4A and 4B), reflecting the intracellular environment and the ensemble kinetic state of the channels at the outset of the experiment. For the G156P trace shown in Fig. 4B, the small current at patch excision suggests most channels were already in the inactivated state; application of LC-CoA pushed channel equilibrium towards the open state, resulting in the large increase in peak current. We note that PIP_2_ becomes incorporated into the membrane over time whereas LC-CoA has higher water solubility and can be easily washed out [29], [40], [43]. The stabilizing effect of PIP_2_ on channel open state thus persisted even after PIP_2_ was removed from the bath solution (Fig. 4A). In contrast, removal of LC-CoA from bath solution resulted in an apparent decrease in peak current amplitude and increase in inactivation rate of G156P patches previously exposed to LC-CoA (Fig. 4B). In addition, stable channel activation was frequently not sustained over longer exposures to LC-CoA (Fig. 4B), and channel current increased little during LC-CoA application when channel currents inactivated to a steady-state level (not shown). This is in marked contrast to other inactivating mutations, such as the intracellular domain mutation R192E, which activates to previous peak responses with applied LC-CoA after inactivation current decay plateaued (Fig. 4B, lower trace). Thus, G156P channels have an inactivation defect that is less sensitive to recovery by channel activators PIP_2_ and LC-CoA.

**Figure 5:**
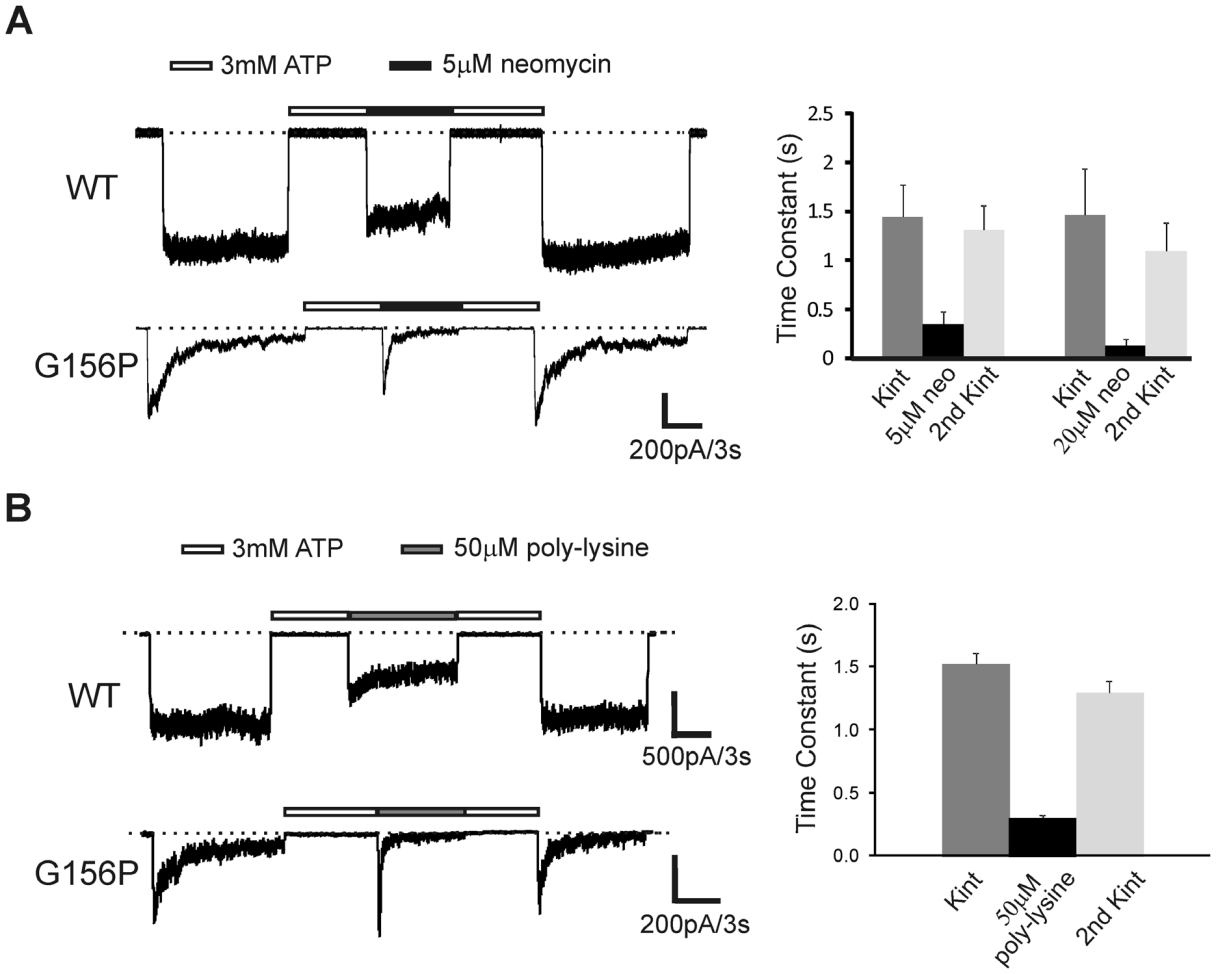
G156P inactivation rate is increased by neomycin or poly-lysine. (A) *Left*, example traces of G156P channels exposed to Kint or Kint with 5 µM neomycin upon ATP recovery of channels. *Right*, summary bar plot of mean decay time constant ± S.E.M. (n = 7–8) for mono-exponential function fit to decay currents during the first Kint exposure, the neomycin exposure, and the subsequent second exposure to Kint. (B) The same as in (A) except that patches were exposed to 50 µM poly-lysine to sequester PIP_2_ (n = 6). For both 5 and 20 µM neomycin as well as 50 µM poly-lysine, the inactivation time constants are significantly shorter than those observed in the initial or subsequent Kint exposures (*p*<0.001, one-way ANOVA with Tukey’s post-hoc test); whereas no significant difference is observed between the initial and subsequent Kint exposures.

If G156P channel inactivation is a consequence of decreased open state stability, and an increased membrane pool of PIP_2_ favors the channel toward the open state, then inhibiting PIP_2_ from binding with the channel would predictably decrease peak activation amplitudes and accelerate inactivation. To test this, we perfused WT and G156P inactivation mutant channel patches with neomycin, which binds PIP_2_ [44] and has been used in many studies to reversibly inhibit PIP_2_-channel interactions [45]–[49]. Application of 5 µM and 20 µM neomycin increased inactivation rate in a dose dependent manner, quantified as a decrease in the decay constant of a mono-exponential fit to the current decay (Fig. 5A). R314A channel inactivation also increased with 20 µM neomycin (data not shown). Application of another widely used PIP_2_ scavenger poly-L-lysine (50 µM; M.W. 500–2,000, Sigma) [7], [50], [51] likewise increased the rate of inactivation in G156P channels (Fig. 5B). Increased rates in the presence of neomycin or poly-L-lysine indicate that channel closure caused by inactivation increases as a result of reduced apparent PIP_2_ affinity. Collectively, the PIP_2_ and neomycin/poly-L-lysine results show that mutant channel sensitivity to PIP_2_ determines the rate of inactivation.

### Gain-of-Function Mutations Stabilize Inactivation

**Figure 6:**
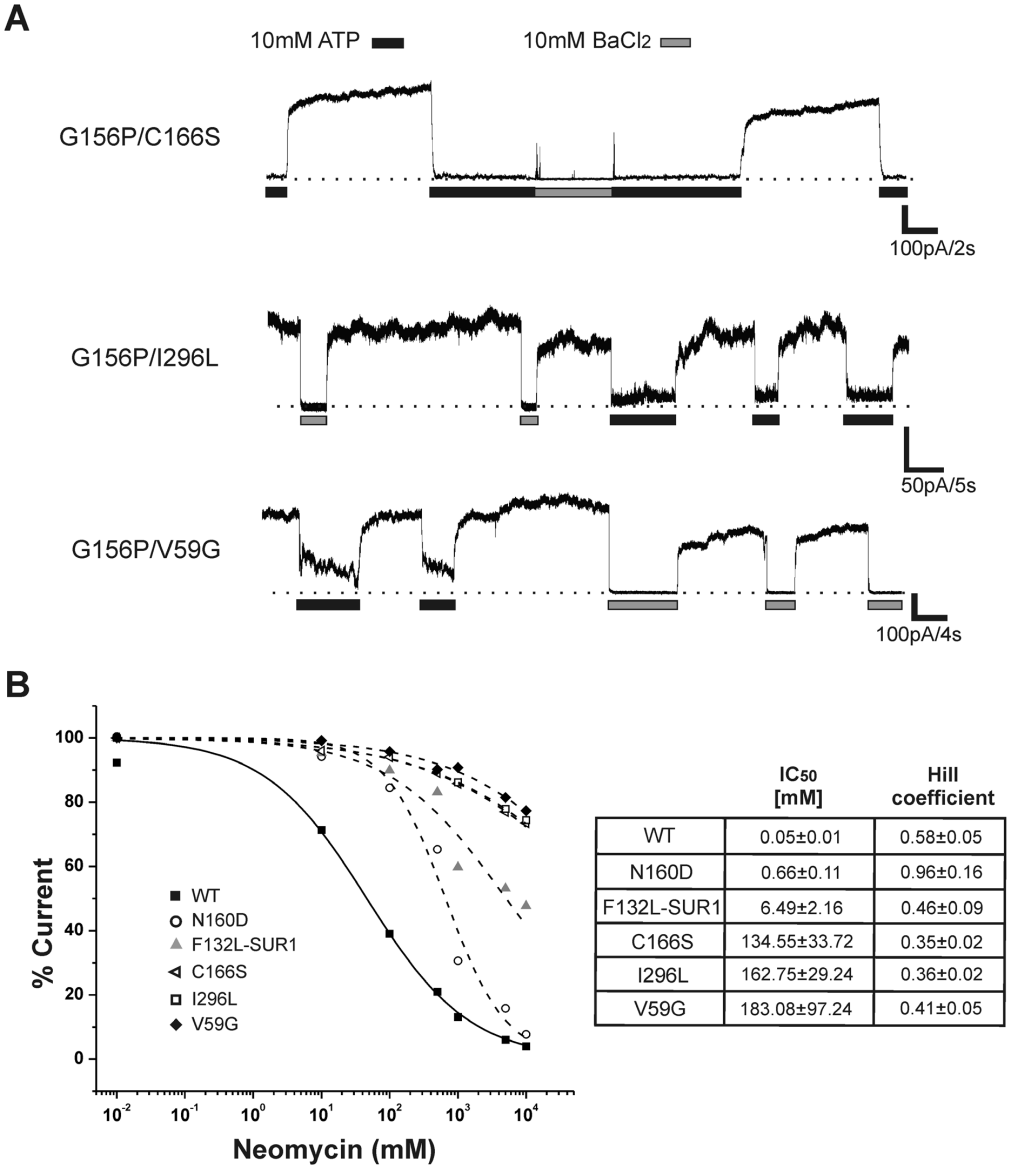
Increasing open-state stability eliminates inactivation. (A) Sample traces from double mutant channels containing G156P and a second disease gain-of-function mutation. Outward currents were recorded at +50 mV. BaCl_2_ blocked K_ATP_ currents to establish baseline. Spikes between different solutions in the middle trace are solution exchange artifacts. (B) Neomycin dose-response curves for WT (solid line) and the various gain-of-function mutations (dashed lines). IC_50_ values listed on the right were obtained by fitting the curves with the following equation: % current = 100×(1/(1+([neomycin]/IC_50_)^H^), where % current is the current observed at a specific neomycin concentration ([neomycin]) expressed as the percentage of that observed in Kint, and H is the Hill coefficient.

Numerous gain-of-function mutations in Kir6.2 and SUR1 have been reported [8], many of which cause neonatal diabetes [52]. We tried to stabilize the inactivating channel in the open state by introducing transmembrane and cytoplasmic domain mutations shown to cause K_ATP_ channel overactivity by increasing apparent PIP_2_ affinity, thus the open probability of the channel. Mutations from Kir6.2 N-terminal cytoplasmic domain (V59G) [53], the second transmembrane lining segment (N160D and C166S) [54], [55], G-loop in the C-terminal cytoplasmic domain (I296L) [56], and TMD0 of SUR1 (F132L) [57] were placed in a Kir6.2 G156P background. Inside-out patch recordings at +50 mV membrane potential produced stable outward currents that were blocked completely by 10 mM BaCl_2_ (Fig. 6A). Currents were blocked partially (for G156P/V59G) or nearly completely (for G156P/C166S and G156P/I296L) by 10 mM ATP similar to channels that only contained gain-of-function mutations reported in previous studies [53], [54], [56]. These results show that inactivation is overcome by shifting the closed-to-open equilibrium dramatically towards the open state, much like the effect of increasing membrane PIP_2_ concentrations. Interestingly, the double mutations Kir6.2-G156P/N160D and Kir6.2-G156P+SUR1-F132L still inactivated (data not shown). Exceptions to rescue by mutations could be explained by considering the degree of enhancement of open state stability by the different gain-of-function mutations. To directly test this, we assessed the apparent PIP_2_ affinity of channels containing the various gain-of-function mutations by constructing dose-response curves to neomycin, which inhibits channel activity by competing for interactions with PIP_2_ (see Fig. 5A). As shown in Fig. 6B, the IC_50_ values for neomycin have the following order: WT<N160D<SUR1-F132L<<C166S <I296L<V59G. These results are consistent with the notion that the Kir6.2-N160D and SUR1-F132L mutations failed to rescue G156P inactivation because they have relatively lower apparent affinity for PIP_2_ compare to V59G, C166S and I296L which did rescue G156P inactivation (the neomycin IC_50_ values for N160D and SUR1-F132L are at least 20-fold lower than those for C166S, I296L and V59G).

## Discussion

In this report, we describe a novel mutation in the pore domain of Kir6.2, G156P that causes K_ATP_ channel inactivation. Several lines of evidence suggest that the inactivation observed in the mutant channel results from destabilization of a PIP_2_-controlled gate near the cytoplasmic end of TM2. First, increasing PIP_2_ or LC-CoA concentrations that stabilize the PIP_2_-controlled gate reverses G156P inactivation. Second, sequestering PIP_2_ by neomycin or poly-lysine increases the rate of G156P inactivation. Third, combination of G156P with activating mutations known to have increased open state stability due to increased apparent PIP_2_ affinity alleviates or eliminates inactivation. These results led us to conclude that proline mutation of the central glycine in TM2 of Kir6.2 decreases open state stability by disrupting K_ATP_ gating induced by PIP_2_ binding. To our knowledge, this is the first report of a Kir pore mutation that causes inactivation by destabilizing opening of the PIP_2_-controlled gate.

### Mechanism of G156P Channel Inactivation and Reactivation

**Figure 7:**
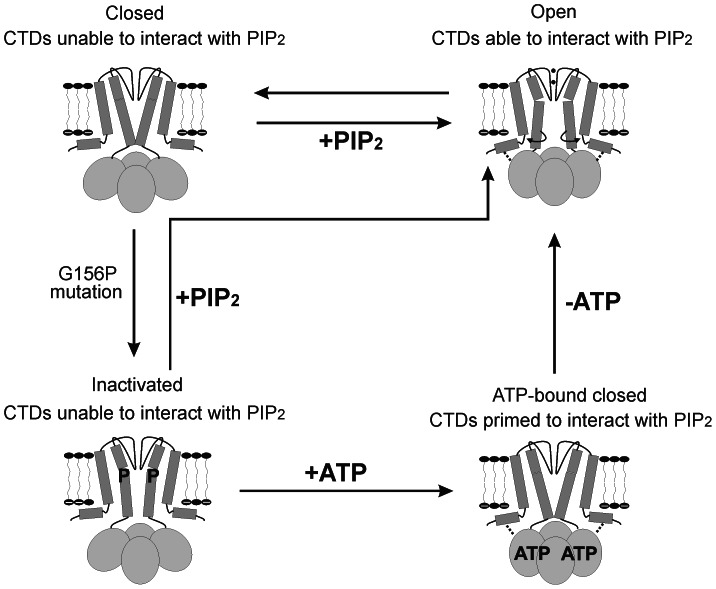
Cartoon depiction of G156P inactivation. WT channels can transition from closed to open state via a change in the cytoplasmic domain (CTD) conformation (indicated by the dotted lines that link the slide helix with the CTDs) with concomitant bending of TM2 near G156 as well as rotation of the lower TM2 that opens the gate near the helix bundle crossing (model based on Bavro et al. [14]). Proline mutation G156P causes a transition into an inactivated state; limited mobility in TM segments favors a closed bundle crossing conformation, perhaps by preventing TM2 rotation and/or conformational transition at the CTDs. Increased PIP_2_ levels can sustain channel activity by stabilizing the CTDs and TM2 in the open conformation. ATP, which binds at the interface of the CTDs, likely induces a conformational change that recovers coupling at the TM-CTD interface (bottom right). Removing ATP allows channels to revisit a state in which PIP_2_ can readily re-bind to activate the channel, if only briefly.

In K_ATP_ channels, the prevailing model assigns the TM2 helix bundle crossing as the “ligand-sensitive” gate [8], [14], [15], [17], [58]. PIP_2_ stabilizes opening of the gate by interacting with the Kir6.2 cytoplasmic domains; whereas ATP, which also interacts with Kir6.2 cytoplasmic domains stabilizes closure of the gate. As illustrated in the cartoon shown in Fig. 7, we propose that binding of intracellular ligands to the cytoplasmic domain induces a conformational change that leads to propagation of a gating signal to the channel pore TMs. We hypothesize that conformational constraint placed on TM2 by G156P attenuates coupling between the TM and cytoplasmic domains and leads to channel inactivation. PIP_2_ binding or gain-of-function mutations stabilize the channel in the open state to prevent inactivation, and ATP binding induces conformational changes in the cytoplasmic domains that transiently recover the functional link between Kir6.2 TM-cytoplasmic domains, priming channels for reactivation.

Transduction of ligand binding to channel gating is a reversible process, and mutations altering any structural component involved in binding, transduction, and/or gating could affect the entire allosteric pathway. If TM2 moves dynamically to open and close the PIP_2_-controlled gate, it would be expected that mutations which inhibit TM2 movements may also affect PIP_2_ response. Substitution of the glycine with a proline likely kinks the helix in a direction unfavorable for TM2 bending that is necessary for the channel to fluctuate between open and closed states. The inactivation phenotype emerges by shifting channel equilibrium away from the PIP_2_-bound open state towards a closed state that is unable to recover. Manipulations that stabilize channel-PIP_2_ interactions (Fig. 4 and 6) shift the equilibrium in the opposite direction to prevent or reverse inactivation.

An important finding from this study is that inactivated G156P channels, upon ATP exposure, are reactivated when ATP is subsequently removed. ATP could serve as a substrate for lipid kinases present in the membrane patch thus replenishing membrane PIP_2_ and reactivating mutant channels. However, this is unlikely to be a major mechanism since all recordings were performed in a solution containing 1 mM EDTA to chelate Mg^2+^, a cofactor required for lipid kinases. Further supporting this, a non-hydrolyzable ATP analogue, AMP-PNP, also reactivated the mutant channel. We therefore propose that ATP binding induces a conformational change that realigns the cytoplasmic and transmembrane domains to allow channels to interact with PIP_2_ and access the open state before inactivating again. Through mass action ATP would pool channels into ATP-bound closed states away from the inactivated state; thus when ATP is removed, channels could transition through the unbound closed state to briefly occupy the PIP_2_-bound open state. This is consistent with the relationship between the extent of recovery and the concentration and duration of ATP exposure.

### Towards Understanding the Structural Basis of K_ATP_ Channel Gating–lessons Learned from Inactivation Mutations

Mutations which alter gating kinetics may offer insight into gating mechanisms. In addition to the G156P mutation described here, we have previously reported several mutations that cause similar inactivation in K_ATP_ channels [25], [26], [29]. These mutations are located in different parts of the channel protein complex and each provides a unique perspective on the structural connections required to maintain stable channel activity. Mutations of residues in the cytoplasmic domain of Kir6.2 near the Kir6.2 subunit-subunit interface, including R192, E292, R301 and R314 [25], [26], cause inactivation by disrupting inter-Kir6.2 subunit cytoplasmic domain interactions required to maintain stable interactions with PIP_2_ [26]. Mutation E128W in the second cytoplasmic loop of the first transmembrane domain of SUR1 also leads to channel inactivation [29], in this case by disrupting the interaction between SUR1 and Kir6.2 that is necessary for stable channel-PIP_2_ interactions [29]. Finally, the G156P mutation likely imposes physical constraint on TM2 to prevent conformational coupling between the pore domain and the cytoplasmic domain upon binding of PIP_2_, thus trapping the channel in an inactivated, non-open state [14]. Collectively, these inactivation mutations suggest that sustained high spontaneous *P_o_* in K_ATP_ channels requires inter-subunit interactions at the interfaces of Kir6.2 and Kir6.2-SUR1 cytoplasmic domains near the membrane to mediate PIP_2_ interactions as well as coupling between the cytoplasmic domain and the transmembrane pore domain of Kir6.2 to propagate the gating effect of PIP_2_. Consistent with this view, several recent structural studies have highlighted the importance of the coupling between the cytoplasmic domains near the membrane and the transmembrane domains in Kir channel gating. For example, a series of KirBac structures in various states of the gating process have revealed conformational changes in the cytoplasmic domains involving structural rearrangements at the subunit-subunit interface and movement of the slide helix near the membrane that are coupled to ion configuration at the selectivity filter and the diameter of the cytoplasmic end of the pore [59]. Moreover, crystal structures of Kir2 and Kir3 channels in complex with PIP_2_ have shown that PIP_2_ binds at an interface between the transmembrane domain and the cytoplasmic domain and causes the cytoplasmic domains to be tethered to the transmembrane domain to widen the opening at the helix bundle crossing and/or at another constriction point below called the G-loop [15], [17].

A common feature of all the K_ATP_ inactivation mutants is that ATP recovers channels from inactivation. As the ATP binding pocket is located at the cytoplasmic Kir6.2 subunit interface near the membrane, it is likely that binding of ATP facilitates subunit-subunit interactions and the coupling between the cytoplasmic domain and the pore domain to allow PIP_2_-mediated gating to occur transiently when the inhibitory effect of ATP is removed. Interestingly, our results show that the G156P mutant exhibited faster inactivation and lower sensitivity to activating ligand, as apparent from the inability of LC-CoA to sustain G156P current (Fig. 4B). However, inactivated G156P channels responded significantly faster to the recovery effect of a given ATP concentration than the cytoplasmic domain inactivation mutations (Fig. 2C). One possible explanation is that the cytoplasmic domain mutations might affect the ATP binding site coordinated by cytoplasmic domains from adjacent subunits thereby slow down recovery of channels from inactivation. The ATP binding site in G156P channels would be expected to be largely intact. Also worth noting, the ATP-induced recovery is greatly facilitated by the presence of SUR1 in G156P (Fig. 1B) and in other Kir6.2 inactivation mutants [26], consistent with the role of SUR1 in facilitating Kir6.2-PIP_2_ interactions [29].

In summary, the study presented here identifies a novel inactivation mutation in the Kir6.2 pore domain that destabilizes the PIP_2_-controlled gate. Because channel-PIP_2_ interactions in this mutant would be expected to be state-dependent and in the context of the complete channel complex, it would be difficult to demonstrate reduced PIP_2_ interactions using currently available biochemical methods involving the use of isolated recombinant channel domains [46], [60]. However, as crystal structures of mammalian Kir channels in complex with PIP_2_ have recently been solved [15], [17], future structural studies may help test our model further. Also, mutation of the equivalent glycine in GIRK to proline has previously been shown to result in greatly reduced basal current measured by two-electrode voltage clamp recording in oocytes, although the study did not examine the kinetics of the mutant channel after activation by Gβγ [58]. It would be interesting to determine in the future if open state stability in other Kir channels share similar structural requirements.

## Supporting Information

Table S1 **G156 mutant channel properties.**
(DOCX)
