# Supplementary material for: A Kir6.2 Pore Mutation Causes Inactivation of ATP-Sensitive Potassium Channels by Disrupting PIP2-Dependent Gating
Source: PLoS One. 2013 May 20;8(5):e63733. doi: 10.1371/journal.pone.0063733 (PMC3659044; doi:10.1371/journal.pone.0063733)
Supplement: Table S1 — G156 mutant channel properties. (DOCX) [file pone.0063733.s001.docx]

**Table S1. G156 Mutant Channel Properties**

| **Channel**^1^ | **Patch activity**^2^ | **Number of patches tested** | **Inactivation** |
| --- | --- | --- | --- |
| G156(WT) | Yes | >20 | No |
| G156A | Yes | 7 | No |
| G156C | Yes | 11 | Mild |
| G156D | No | 9 | N/A |
| G156E | Yes | N/A | No |
| G156H | No | 7 | N/A |
| G156I | No | 5 | N/A |
| G156K | No | - | N/A |
| G156L | No | 5 | N/A |
| G156M | No | 5 | N/A |
| G156N | Yes | 4 | Mild |
| G156P | Yes | >20 | Severe |
| G156Q | No | 3 | N/A |
| G156R | No^4^ | - | N/A |
| G156S | Yes | 8 | No |
| G156T | Yes | 5 | Mild |
| G156V | No | 7 | N/A |
| G156W | No | 6 | N/A |
| G156Y | No | 5 | N/A |

^1^ G156 was substituted with all other amino acids with the exception of phenylalanine, which is a bulky residue and expected to abolish channel activity based on phenotype of other mutants with amino acids of similar properties at the position.

^2^ All recordings were made in Kint solution containing 1mM EDTA at -50mV membrane potential.
